# Supplementary material for: Splenic red pulp macrophages eliminate the liver-resistant Streptococcus pneumoniae from the blood circulation of mice
Source: Sci Adv. 2025 Mar 12;11(11):eadq6399. doi: 10.1126/sciadv.adq6399 (PMC11900858; doi:10.1126/sciadv.adq6399)
Supplement: Supplementary file 1 — Figs. S1 to S9 Tables S1 to S5 Legends for movies S1 to S5 [file sciadv.adq6399_sm.pdf]

Science Advances
AAAS

Supplementary Materials for

Splenic red pulp macrophages eliminate the liver-resistant *Streptococcus pneumoniae* from the blood circulation of mice

Haoran An *et al.*

Corresponding author: Haoran An, ahr@bjmu.edu.cn; Qionghai Dai, daiqh@tsinghua.edu.cn; Jing-Ren Zhang, zhanglab@tsinghua.edu.cn

Sci. Adv. **11**, eadq6399 (2025)
DOI: 10.1126/sciadv.adq6399

The PDF file includes:

Figs. S1 to S9
Tables S1 to S5
Legends for movies S1 to S5

Other Supplementary Material for this manuscript includes the following:

Movies S1 to S5

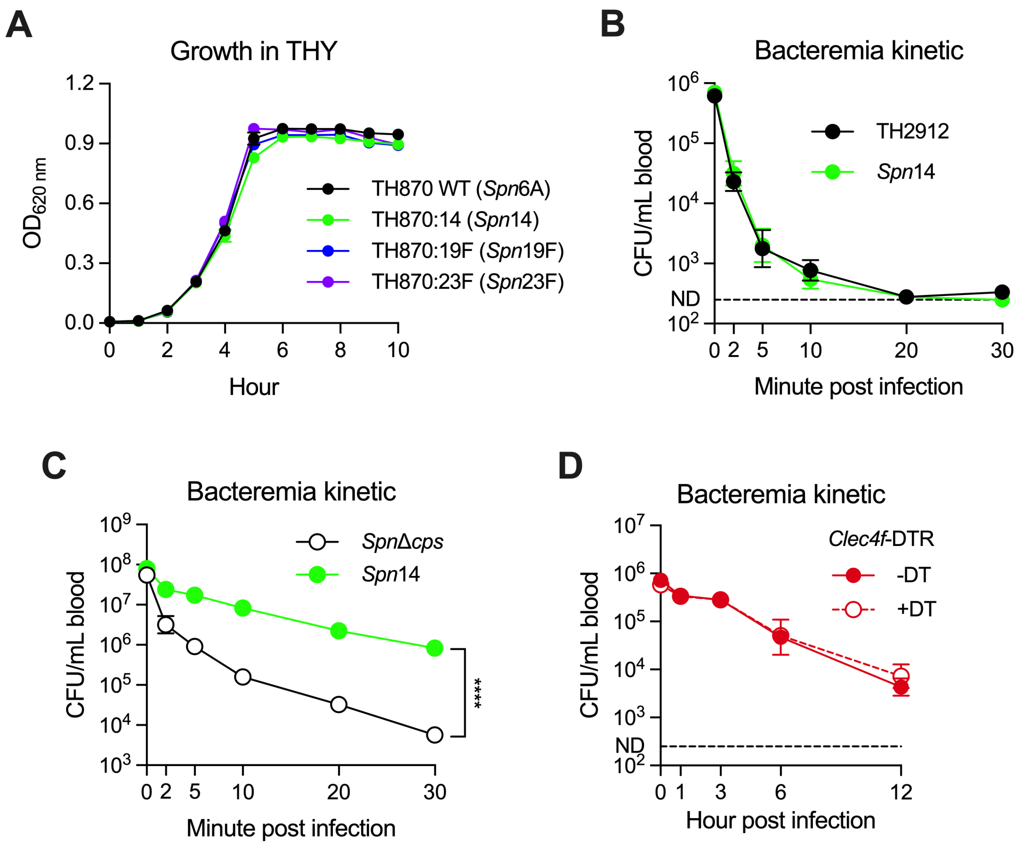

Fig. S1. Clearance of *S. pneumoniae* strains from the bloodstream of mice. (A) Growth curve of *S. pneumoniae* strains in THY medium. The *in vitro* growth of isogenic capsule-switched strains in TH870 background was compared. **(B)** Comparison of the bacteremia kinetics of the clinical serotype-14 (TH2912) and capsule-switched (*Spn*14) strains during the first 30 min post i.v. infection with 10^6 CFU per mouse. n = 3. **(C)** The production of heterologous capsule in the capsule-switched strain as reflected by higher bacterial loads of *Spn*14 than the unencapsulated TH870Δ*cps* during the first 30 min post i.v. infection with 10^8 CFU per mouse. n = 3-6. **(D)** Dispensable role of liver KCs for the clearance of HV pneumococci. Bacteremia kinetics were monitored in KC-specific depletion *Clec4f*-DTR mice (+DT) and control mice (-DT) post i.v. infection with 10^6 CFU of the *Spn*6A. n = 3. Significance was compared by two-way ANOVA test (C). **** $P < 0.0001$.

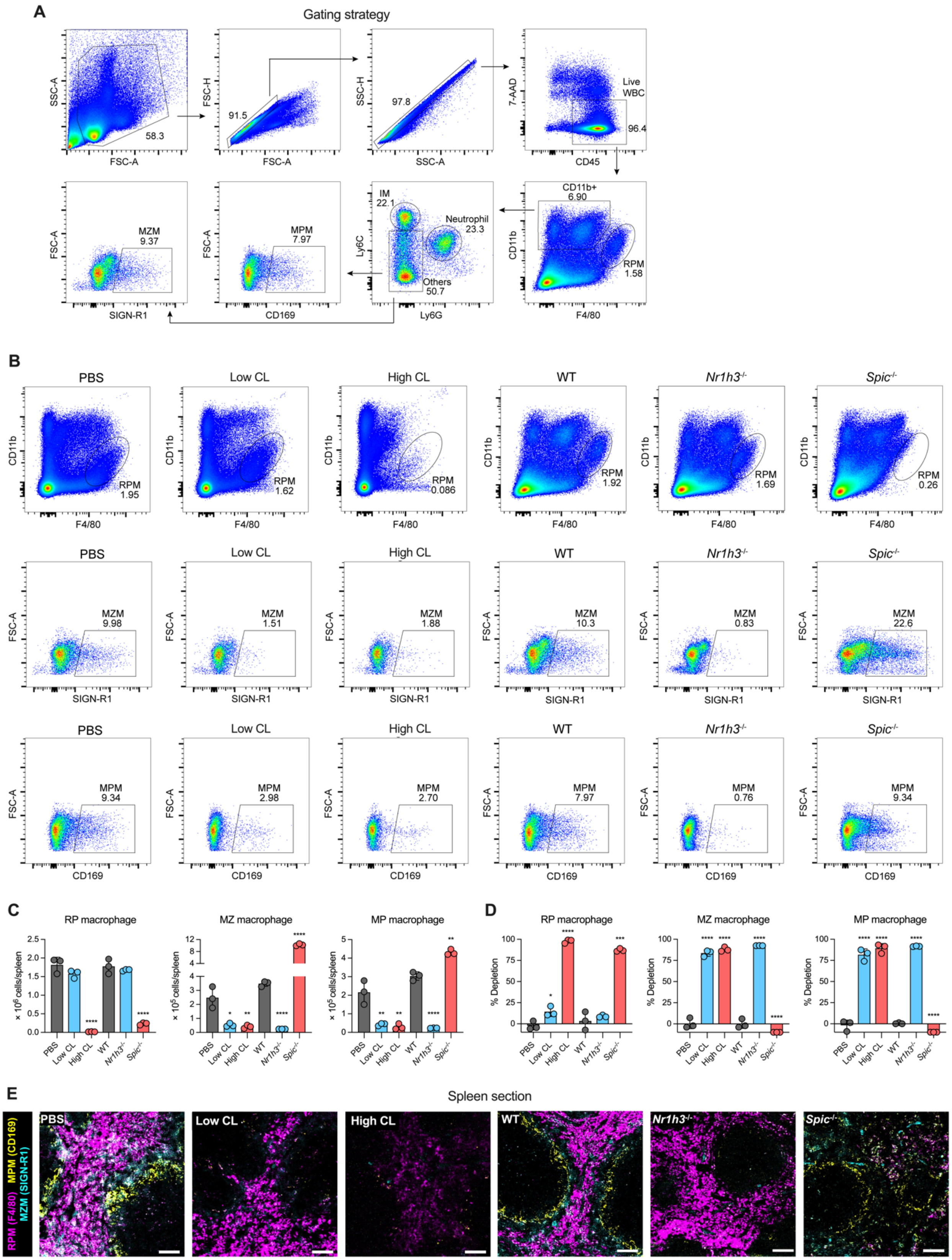
A
Gating strategy
SSC-A
FSC-A
58.3
FSC-H
91.5
SSC-H
97.8
SSC-A
7-AAD
Live WBC
96.4
CD45
CD11b+
6.90
CD11b
RPM
1.58
F4/80
IM
22.1
Neutrophil
23.3
Ly6C
Others
50.7
Ly6G
MPM
7.97
CD169
MZM
9.37
SIGN-R1
B
PBS
Low CL
High CL
WT
Nr1h3-/-
Spic-/-
RPM 1.95
RPM 1.62
RPM 0.086
RPM 1.92
RPM 1.69
RPM 0.26
MZM 9.98
MZM 1.51
MZM 1.88
MZM 10.3
MZM 0.83
MZM 22.6
MPM 9.34
MPM 2.98
MPM 2.70
MPM 7.97
MPM 0.76
MPM 9.34
C
RP macrophage
MZ macrophage
MP macrophage
× 10^6 cells/spleen
× 10^5 cells/spleen
D
% Depletion
E
Spleen section
RPM (F4/80) MPM (CD169) MZM (SIGN-R1)
PBS
Low CL
High CL
WT
Nr1h3-/-
Spic-/-

Fig. S2. Validation of selective depletion methods targeting splenic macrophage populations. **(A)** Gating strategy for analyzing splenic phagocytes using flow cytometry. Live immune cells ($CD45^{+}$/7-AAD^{-}) in the spleen were identified as RP macrophages (RPM, $CD11b^{low}$/$F4/80^{+}$), neutrophils ($CD11b^{+}$/$Ly6C^{+}$/$Ly6G^{+}$), inflammatory monocytes (IM, $CD11b^{+}$/$Ly6C^{high}$/$Ly6G^{-}$), MZ macrophages (MZM, $CD11b^{+}$/$F4/80^{-}$/SIGN-$R1^{+}$), and MP macrophages (MPM, $CD11b^{+}$/$F4/80^{-}$/$CD169^{+}$). **(B)** Representative flow cytometry results to show the depletion of splenic macrophage populations in CL-treated and genetically deficient mice. (**C and D**) Quantification of the depletion efficiency of splenic macrophages. The absolute numbers per spleen (C) and decline ratios (D) of RP, MZ, and MP macrophages were calculated according to the flow cytometry data. (**E**) Representative immunofluorescent staining of spleen sections to show the loss of different macrophage subtypes under the depletion conditions. RP, MZ, and MP macrophages were indicated by staining of AF647 F4/80 (Magenta), AF488 SIGN-R1 (Cyan), and AF594 CD169 (Yellow), respectively. Scale bar, 100 μm. Significance was compared by one-way ANOVA test (C and D). * $P < 0.05$, ** $P < 0.01$, *** $P < 0.001$, **** $P < 0.0001$.

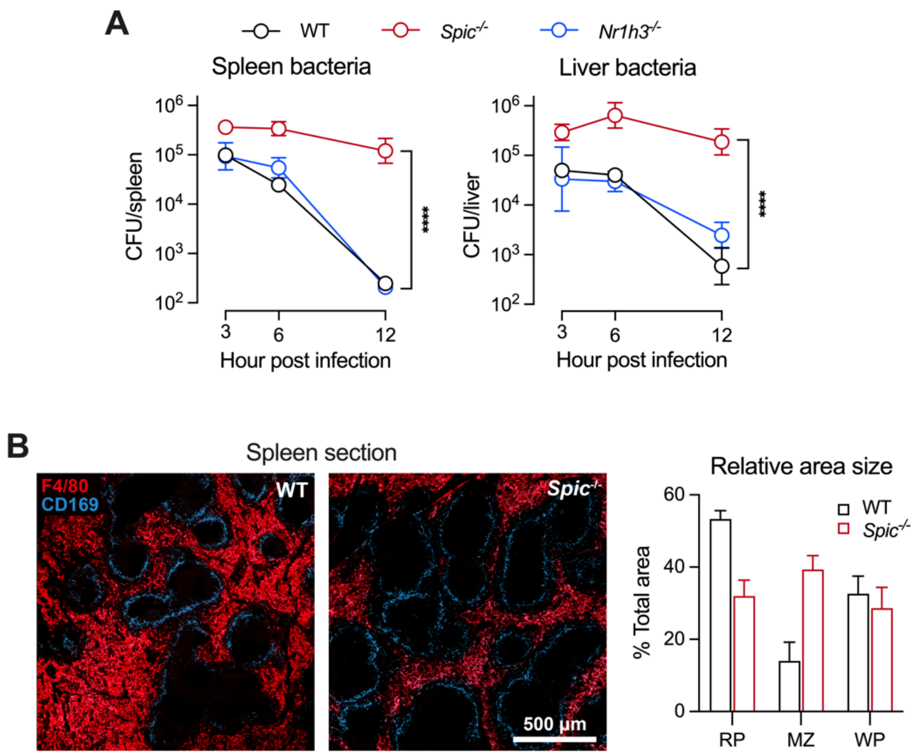

Fig. S3. Essential role of RP macrophages in clearing HV pneumococci. (A) Essential role of RP macrophages in systemic control of HV pneumococci. Bacterial loads in the spleen and liver were counted in *Spic*$^{-/-}$ or *Nr1h3*$^{-/-}$ mice post infection with 10^6 CFU of *Spn*6A. n = 3. **(B)** Representative fluorescence section to show the compartments of the spleen. RP and MZ were indicated by staining of AF647 F4/80 (Red) and AF594 CD169 (Blue), respectively. The WP (dark area) was delimited according to the MZ. Quantification of the area size of each compartment was calculated based on 5 sections from each spleen and shown on the right. n = 3. Significance was compared by two-way ANOVA test (A). **** $P < 0.0001$.

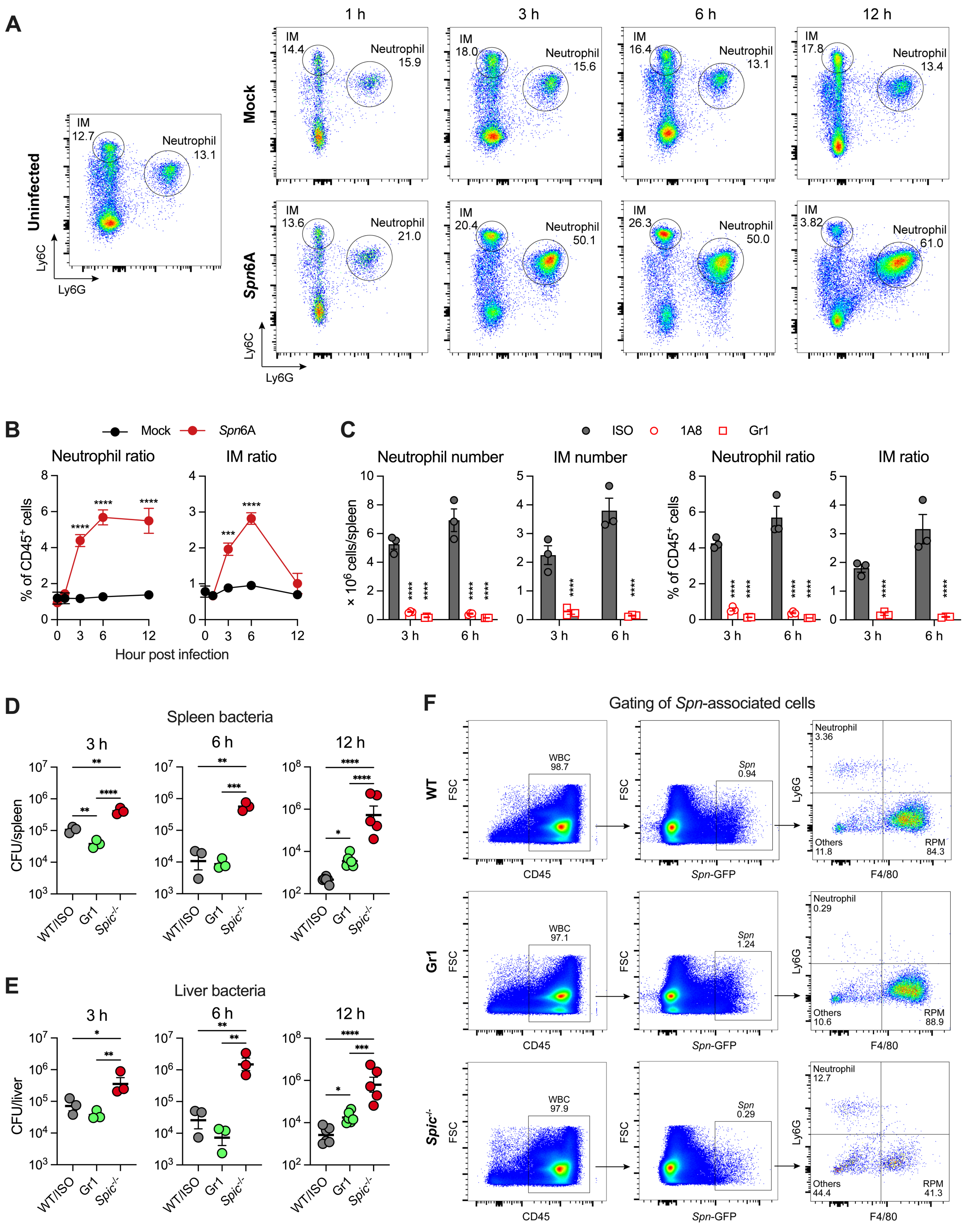

Fig. S4. Dominant role of RP macrophages for the elimination of HV pneumococci in the spleen. (A and B) Representative flow cytometry (A) and quantification (B) results to show the infiltration of neutrophils and IMs in the spleen during pneumococcal infection. The kinetics of

neutrophils and IMs were assessed in the first 12 hrs post i.v. infection with 10^6 CFU of HV *Spn*6A. Mock infected mice were i.v. injected with 100 µl of Ringer's solution. n = 3. **(C)** Prevention of neutrophil and IM recruitment during the "eclipse phase" by antibody-mediated depletion. The mice were i.v. received with two doses of 500 ng 1A8 or Gr1 antibodies at 24 hr and 5 min prior to i.v. infection with 10^6 CFU of HV *Spn*6A. The absolute numbers and ratio of neutrophils and IMs in the spleen were then assessed by flow cytometry. The same dose of isotype control (ISO) antibodies was included as controls. n = 3. **(D and E)** Impact of neutrophil and IM depletion on the systemic clearance of HV pneumococci. Bacterial loads in the spleen (D) and liver (E) were counted in control (WT/ISO), Gr1-treated, and *Spic*$^{-/-}$ mice post infection with 10^6 CFU of *Spn*6A. n = 3-6. **(F)** Representative flow cytometry results to show the distribution of pneumococci in splenic $CD45^+$ cells. Splenocytes were analyzed at 30 min post i.v. infection with 10^7 CFU of GFP-expressing *Spn*6A. Significance was compared by two-way (B) or one-way (C to E) ANOVA test. * $P < 0.05$, ** $P < 0.01$, *** $P < 0.001$, **** $P < 0.0001$.

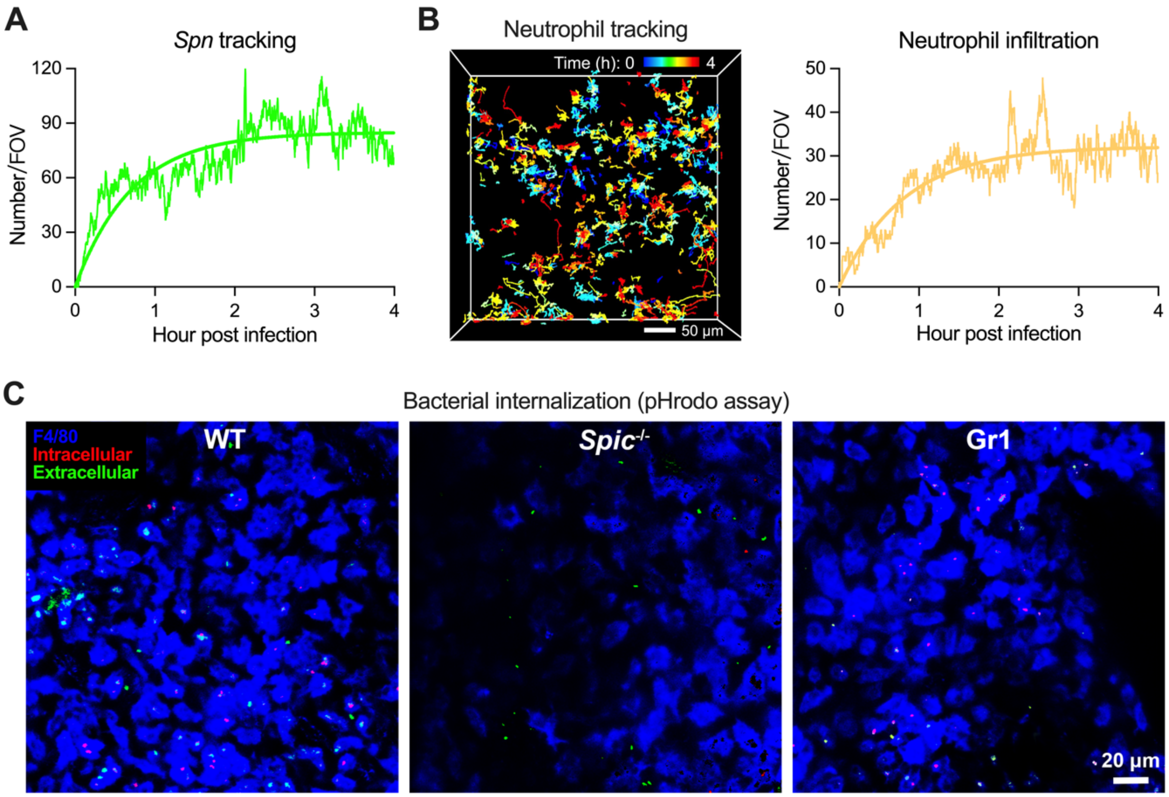

Fig. S5. Analysis of pneumococcal clearance in the spleen by 2pSAM. **(A)** Curve of tracked pneumococcal cells during the first 4 hr post infection with 2×10^7 CFU of GFP-expressing *Spn*6A (Movie S1). **(B)** Overlay of tracked traces (left panel) and curve of the quantity (right panel) of the infiltrated neutrophils during the first 4 hr post infection as in (A). Scale bar, 50 µm. **(C)** Representative 2pSAM images to illustrate the internalization of *Spn*6A in the spleen of WT, *Spic*$^{-/-}$, and Gr1-treated mice. Uptake of *Spn*6A was indicated by the activation of pHrodo Red dye (Inside), whereas outside bacteria remained in green. Images were obtained at 0.5-1 hpi. Scale bar, 20 µm.

A

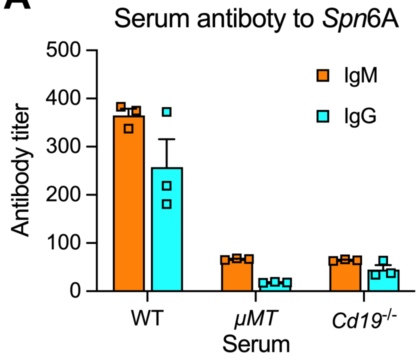

B

IgG receptors
(Primary RPM)

**Protein**	**Abundance**
FcγRI	ND
FcγRIIB	6.3×10^7
FcγRIII	1.0×10^7
FcγRIV	0.8×10^7
FcRn	3.8×10^7

Fig. S6. The essential role of natural antibodies in splenic clearance of HV pneumococci. (A) Detection of anti-pneumococcal natural antibodies in murine serum. Titers of IgM and IgG in serum isolated from WT, *μMT*, and *Cd19*$^{-/-}$ mice were measured by ELISA using *Spn*6A whole cells as antigen. n = 3. **(B)** Relative abundance of known IgG receptors in murine RP macrophages. Data were the average amount of three replicates. ND, not detectable.

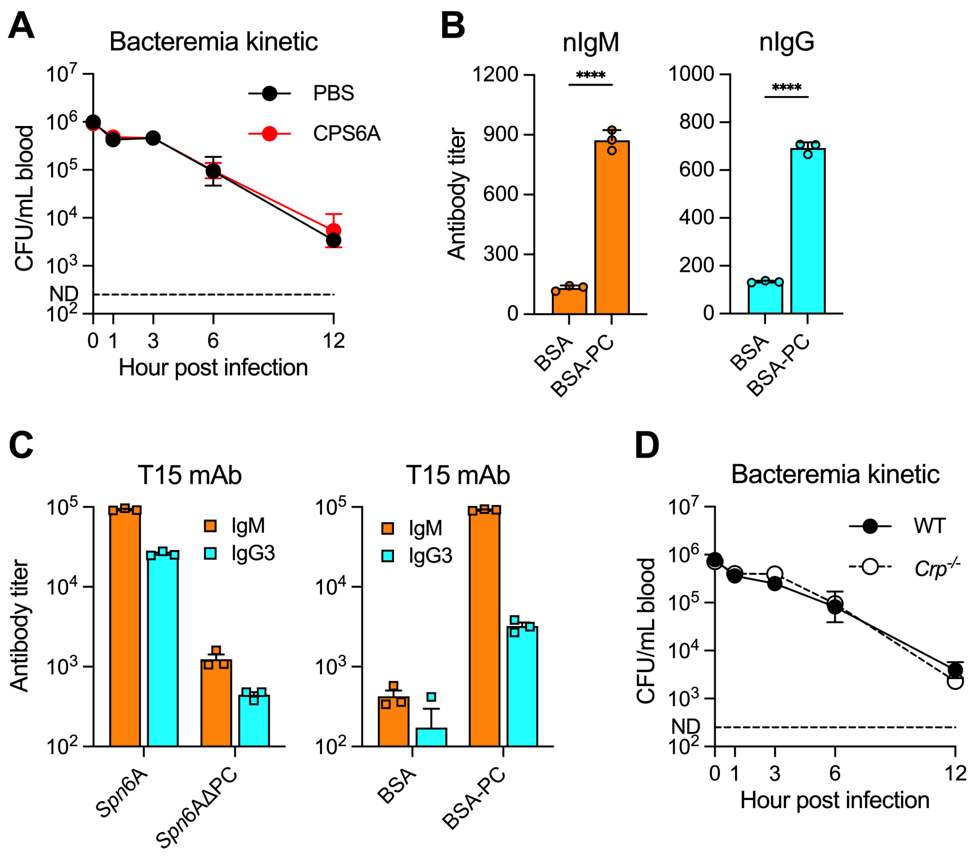

Fig. S7. Pneumococcal cell wall PC as a target antigen of natural antibodies. (A) Irrelevance of capsular polysaccharide in HV pneumococcal clearance. Bacteremia kinetics were monitored in WT mice pre-administrated by PBS or 400 µg serotype 6A capsule (CPS6A) at 5 min before infection with 10^6 CFU *Spn*6A. n = 3. **(B)** Detection of anti-PC antibodies in purified natural antibodies. Purified nIgM and nIgG were diluted at 1 mg/ml in PBS, and titers of IgM and IgG were measured by ELISA using BSA-conjugated PC as antigen. n = 3. **(C)** Verification of the specificity of the recombinant T15 monoclonal anti-PC antibodies. The T15 IgM and IgG3 were diluted at 1 mg/ml in PBS, and titers were measured by ELISA using *Spn*6A whole cells, PC-free *Spn*6A, or BSA-conjugated PC as antigens. n = 3. **(D)** Dispensable role of CRP in HV pneumococcal clearance. Bacteremia kinetics were monitored in WT and *Crp*$^{-/-}$ mice post infection with 10^6 CFU *Spn*6A. n = 5. Significance was compared by student's *t* test (B). **** $P < 0.0001$.

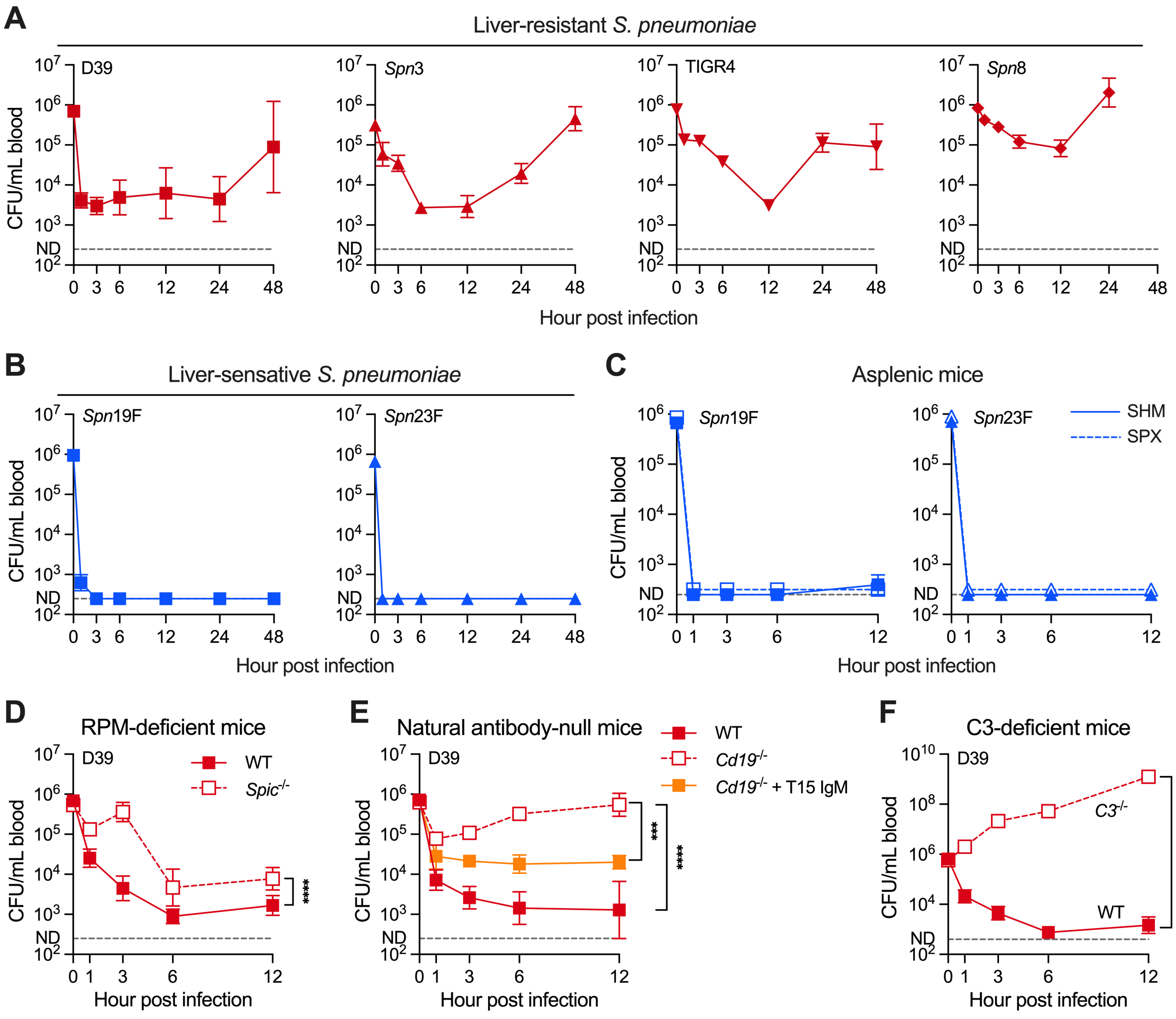

Fig. S8. Serotype-independent splenic immunity against HV pneumococci. (A and B) Bacteremia kinetics in WT mice post i.v. infection with HV (A) and LV (B) pneumococci. Blood bacterial loads were counted post infection with 10^6 CFU of HV serotype 2 (D39), serotype 4 (TIGR4), isogenic serotype 3 and 8 strains (*Spn*3 and *Spn*8), and LV serotype 19F and 23F strains (*Spn*19F and *Spn*23F). n = 3-6. **(C)** Dispensable role of spleen for the blood clearance of LV pneumococci. Blood bacterial loads in SPX mice were counted in the first 12 hr post infection as in (B), and compared with the SHM controls. n = 3. **(D)** Contribution of RP macrophages for the blood clearance of D39. Blood bacterial loads in *Spic*$^{-/-}$ mice were counted in the first 12 hr post infection with 10^6 CFU of D39 and compared with the WT controls. n = 6. **(E)** Promotion of anti-PC antibodies to the blood clearance of D39. Blood bacterial loads in *Cd19*$^{-/-}$ mice were counted in the first 12 hr post infection with 10^6 CFU of the bacteria that untreated or pre-opsonized by 5

μg of T15 IgM. n = 3. **(F)** Pivotal role of complement C3 for the blood clearance of D39. Blood bacterial loads in *C3*$^{-/-}$ mice were counted in the first 12 hr post infection as in (D). n = 3-6. Significance was compared by two-way ANOVA test (D-F). *** $P < 0.001$, **** $P < 0.0001$.

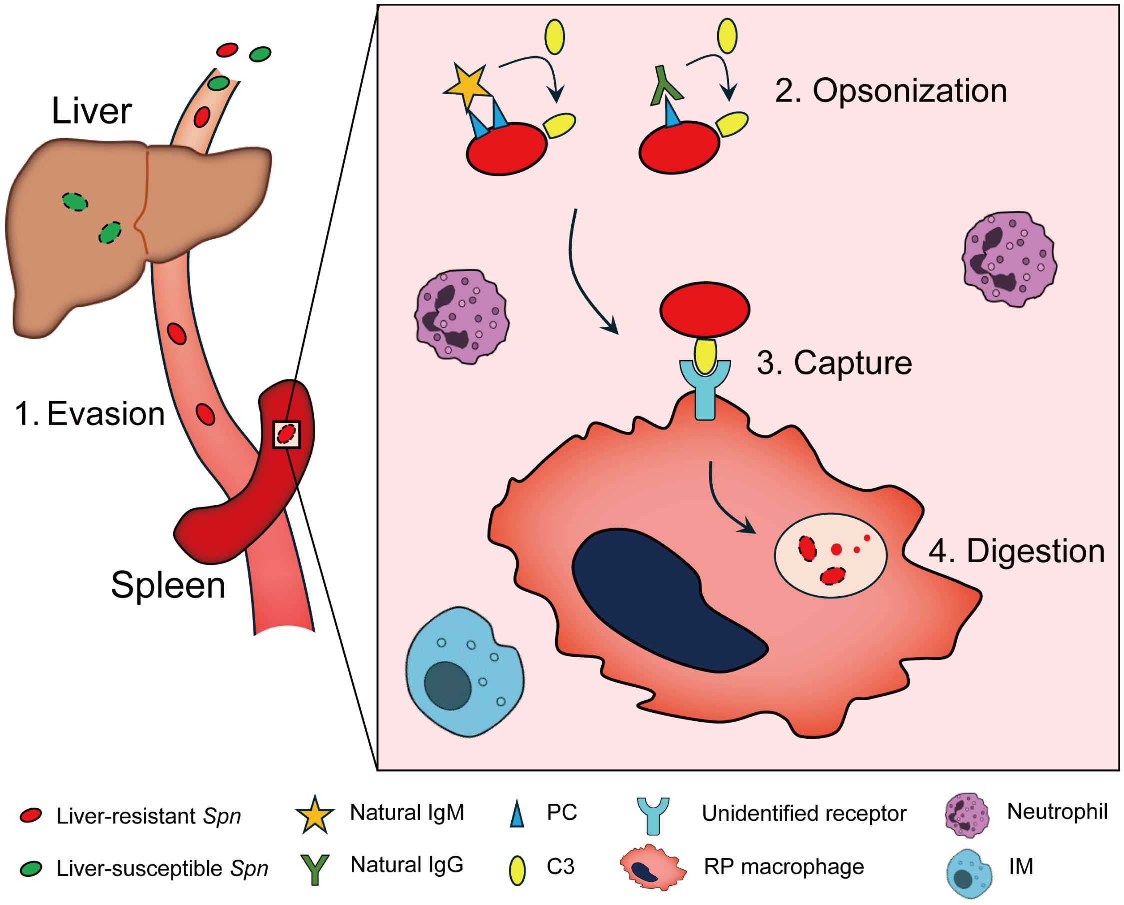

Fig. S9. A model for the RP macrophage-executed immunity against liver-resistant *S. pneumoniae* in the spleen. During bloodstream infection, the LV serotypes of *S. pneumoniae* (liver-susceptible *Spn*) are rapidly eliminated by Kupffer cells in the liver, while the HV serotypes counterparts (liver-resistant *Spn*) evade the hepatic firewall. The spleen acts as the backup to control the liver-resistant *Spn*, in which the RP macrophages are the major effector phagocytes at the early stage of infection. Plasma anti-PC antibodies contribute to the splenic immune defense against liver-resistant *Spn* by promoting complement-dependent bacterial capture and subsequent killing through intracellular digestion.

Table S1. Bacterial strains used in this study.

Strain ID	Species	Serotype	Description
D39		2	Wild type
TIGR4		4	Wild type
TH870		6A	Wild type (*Spn*6A)
TH15921		N/A	Marker-free capsule mutant of TH870 (*Spn*6AΔCPS)
TH15939	*S. pneumoniae*	3	Isogenic serotype 3 strain in TH870 background (*Spn*3)
TH15943		8	Isogenic serotype 8 strain in TH870 background (*Spn*8)
TH15944		14	Isogenic serotype 14 strain in TH870 background (*Spn*14)
TH14188		19F	Isogenic serotype 19F strain in TH870 background (*Spn*19F)
TH15945		23F	Isogenic serotype 23F strain in TH870 background (*Spn*23F)

Table S2. Mouse lines used in this study.

Name	Target	Deficiency	Strain	Source
C3$^{-/-}$	Complement component 3	Complement activation	003641	Jackson Laboratory, Bar Harbor, USA
Nr1h3$^{-/-}$	Nuclear oxysterol receptor LXRα	MZ and MP macrophage development	013761	
µMT	Immunoglobulin heavy chain of IgM	Antibody production	002288	
Ccr2$^{-/-}$	Chemokine (C-C motif) receptor 2	IM recruitment	017586	
Crp$^{-/-}$	C-reactive protein	C-reactive protein	T003305	GemPharmatech, Nanjing, China
C1qa$^{-/-}$	Complement component 1, q subunit, α chain	Classical pathway for C3 activation	T015440	
Fcamr$^{-/-}$	IgA and IgM Fc receptor	IgA and IgM Fc receptor	T029486	
Cd19$^{-/-}$	CD19 antigen	B1 cell development	T012605	
Fcgrt$^{-/-}$	IgG receptor FcRn large subunit p51	Neonatal Fc receptor FcRn	NM-KO-00133	Model Organisms Center, Shanghai, China
Fcmr$^{-/-}$	IgM Fc receptor	IgM Fc receptor	NM-KO-200197	
Spic$^{-/-}$	Transcription factor Spi-C	RP macrophage development	/	Dr. Malay Haldar, University of Pennsylvania
Clec4F-DTR	Diphtheria toxin receptor with promoter of *Clec4F*	Liver KC with DT treatment	/	Dr. Martin Guilliams, Ghent University
Fcgr1/2b/3/4$^{-/-}$	IgG Fc receptor I, IIB, III, IV	IgG FcRI + IIB + III + IV	/	Dr. Jeffery Ravetch, Rockefeller Univeristy
Vsig4$^{-/-}$	V-set and immunoglobulin domain-containing protein 4	Complement receptor CRIg	/	Genentech, CA, USA
Cr2$^{-/-}$	CD21 antigen	Complement receptor CR1/2	T027941	GemPharmatech, Nanjing, China
Itgam$^{-/-}$	Integrin subunit alpha M	Complement receptor CR3	/	Wang et al., 2023 (*48*)
Itgax$^{-/-}$	Integrin subunit alpha X	Complement receptor CR4	/	This study
Itgam$^{-/-}$*Itgax*$^{-/-}$	Integrin subunit alpha M and X	Complement receptor CR3 + CR4	/	

Table S3. Oligonucleotides used in this study.

ID	Sequence (5'-3')
sgRNA	
gRNA15312	GGTGGTGGTTGGAGCACCAA
Primer	
Pr19033	GCTCTAGAGCCACCATGGACTTTTGGCTTTGGTTACTTTACTTC
Pr19034	CCGCTCGAGTCAATGGTGATGGTGATGATGTTGGCATGAAGATCTGGGCCCTGGG
Pr19035	GCTCTAGAGCCACCATGGACCAAGGTGCCCCAGCTAAGCCCAGT
Pr19036	CCGCTCGAGTCAATGGTGATGGTGATGATGGGGTCTTGGGTCATTCTCCAGGACG
Pr19047	CTGGAAATCAAGCGTACGCGTACGGATGCTGCACCAACTGTAT
Pr19048	GCGGCCAAGCTTGGGAGCGGCCGCTCAACACTCATTCCTGTTG
Pr19134	GAGAACCGGTGTACATTCTGAGGT
Pr19135	GAGACTCGAGGCTGAGGAGACGGT
Pr19136	GAGAACCGGTGTACATTCTGACATTG
Pr19137	GAGACGTACGCCGTTTCAGCTCC
Pr19182	GGAATTCATGAAGAACCATTTGCTTTTCTG
Pr19183	GGATGATACATGACCATCCCATAGGGCCGGGATTCTCCTC
Pr19184	GAGGAGAATCCCGGCCCTATGGGATGGTCATGTATCATCC
Pr19185	GAGACTCGAGCAGTCAGTCCTTCCCAAATGT
Pr19186	GAGAAAGCTTGGGAGCGGCCGCTCAATAGCAGGTGCCGCC
Pr19187	GAGACTCGAGCCTCGAGCGCTACAACAAC
Pr19188	GGGCCATGGCGGCCAAGCTT

Table S4. Antibodies used in this study.

Name	Supplier	Catalog number	RRID
AF647 anti-F4/80	Invitrogen	MF48021	AB_10375289
AF488 anti-SIGN-R1	Invitrogen	53-2093-82	AB_2802324
AF594 anti-CD169	BioLegend	142416	AB_2565620
PE anti-F4/80	BioLegend	123110	AB_893486
PE-Cy5 anti-Ly6G	BioLegend	127671	AB_2904289
PE-Cy5 anti-F4/80	Invitrogen	15-4801-82	AB_468798
Anti-Ly6G	Bio X Cell	BE0075-1	AB_1107721
Anti-Ly6C/Ly6G	Bio X Cell	BE0075	AB_10312146
Anti-CD16/32	BioLegend	101302	AB_312800
APC-Cy7 anti-CD45	BD Pharmingen	557659	AB_396774
BV605 anti-CD11b	BioLegend	101257	AB_11126744
FITC anti-F4/80	BioLegend	123108	AB_893500
AF700 anti-Ly6G	Invitrogen	56-9668-82	AB_2802355
eFluor 450 anti-Ly6C	Invitrogen	48-5932-82	AB_10805519
APC anti-SIGN-R1	Invitrogen	17-2093-80	AB_11149350
PE anti-CD169	Invitrogen	12-5755-80	AB_2572624

Table S5. Construction of recombinant plasmids for antibody production.

Plasmid ID	Backbone	Insertion segments	Primers for segments	Template DNA	Digestion
pTH16845	Heavy chain vector for mouse IgM	J chain + VH T15 + CH_{IgM}	Pr19182/Pr19183 (J)	Mouse spleen cDNA	EcoRI/HindIII
			Pr19184/Pr19135 (VH T15)	GenBank: M16334.1	
			Pr19182/Pr19135(J-VH fusion)	J + VH	
			Pr19185/Pr19186(CH_{IgM})	Mouse spleen cDNA	
pTH16838	Heavy chain vector for mouse IgG3	VH T15 + CH_{IgG3}	Pr19134/Pr19135 (VH T15)	GenBank: M16334.1	AgeI/HindIII
			Pr19187/Pr19188 (CH_{IgG3})	Mouse spleen cDNA	
			Pr19134/Pr19188 (VH T15-CH_{IgG3} fusion)	VH + CH_{IgG3}	
pTH14793	Light chain vector for mouse Ig	VL T15 + CL	Pr19136/Pr19137 (VL T15)	GenBank: U29423.1	AgeI/NotI
			Pr19047/Pr19048 (CL)	Mouse spleen cDNA	
			Pr19136/Pr19048 (VL T15-CL fusion)	VL + CL	
pTH16693	pCDH	Mouse Fcmr	Pr19033/Pr19034	Mouse spleen cDNA	XbaI/XhoI
pTH16694	pCDH	Mouse Fcamr	Pr19035/Pr19036	Mouse spleen cDNA	XbaI/XhoI

Movie S1. Long-term illustration of *Spn*6A sequestration in the spleen of WT mouse by 2pSAM. GFP-expressing bacteria (green) were i.v. inoculated at 2 × 10^7 CFU for real-time visualization in the first 4 hr. RP macrophages and neutrophils were stained with PE anti-F4/80 (magenta) and PE-Cy5 anti-Ly6G (yellow), respectively.

Movie S2. Comparison of *Spn*6A capture in the spleen of WT, *Spic*$^{-/-}$, and Gr1-treated mice. Pneumococcal capture was monitored in the first 1 hr post i.v. inoculation with 5 × 10^6 CFU bacteria in WT, RP macrophage-deficient (*Spic*$^{-/-}$), and neutrophil-depleted (Gr1) mice.

Movie S3. Illustration of neutrophil-dependent and -independent elimination of RP macrophage-captured *Spn*6A. Representative views were shown to illustrate neutrophil-dependent (**A**) and independent (**B**) elimination of pneumococci.

Movie S4. Essential role of natural antibodies in *Spn*6A clearance in the spleen. Pneumococcal capture was monitored in the first 1 hr post i.v. inoculation with 5 × 10^6 CFU native (**A**) and 50 µg nIgM-opsonized (**B**) bacteria in *Cd19*$^{-/-}$ mice.

Movie S5. Promotion of *Spn*6A capture in the spleen by monoclonal anti-PC IgM. Pneumococcal capture was monitored in the first 1 hr post i.v. inoculation with 5 × 10^6 CFU native and 25 µg T15 IgM-opsonized bacteria in *Cd19*$^{-/-}$ mice.
